# Mechanism of allele-selective inhibition of huntingtin expression by duplex RNAs that target CAG repeats: function through the RNAi pathway

**DOI:** 10.1093/nar/gks907

**Published:** 2012-10-05

**Authors:** Jiaxin Hu, Jing Liu, Dongbo Yu, Yongjun Chu, David R. Corey

**Affiliations:** Departments of Pharmacology and Biochemistry, UT Southwestern Medical Center at Dallas, Dallas, TX 75390-9041, USA

## Abstract

Huntington’s disease is an incurable neurodegenerative disorder caused by expansion of a CAG trinucleotide repeat within one allele of the huntingtin (*HTT*) gene. Agents that block expression of mutant HTT and preserve expression of wild-type HTT target the cause of the disease and are an alternative for therapy. We have previously demonstrated that mismatch-containing duplex RNAs complementary to the expanded trinucleotide repeat are potent and allele-selective inhibitors of mutant HTT expression, but the mechanism of allele selectivity was not explored. We now report that anti-CAG duplex RNA preferentially recruits argonaute 2 (AGO2) to mutant rather than wild-type *HTT* mRNA. Efficient inhibition of mutant HTT protein expression requires less AGO2 than needed for inhibiting wild-type expression. In contrast, inhibiting the expression of mutant HTT protein is highly sensitive to reduced expression of GW182 (TNRC6A) and its two paralogs, a protein family associated with miRNA action. Allele-selective inhibition may involve cooperative binding of multiple protein–RNA complexes to the expanded repeat. These data suggest that allele-selective inhibition proceeds through an RNA interference pathway similar to that used by miRNAs and that discrimination between mutant and wild-type alleles of *HTT* mRNA is highly sensitive to the pool of AGO2 and GW182 family proteins inside cells.

## INTRODUCTION

Huntington’s disease (HD) is a neurological disorder that afflicts 5–10 per 100 000 individuals in Europe and North America (1–3). HD symptoms typically present in middle age and worsen until death. There are currently no curative therapies and development of therapies to delay the onset of HD or slow its progression remains a major clinical need (4).

HD is caused by an expansion of a CAG trinucleotide repeat within the gene encoding huntingtin (HTT) protein (5). The mutation is autosomal dominant, with wild-type alleles having 6–34 repeats and mutant alleles containing 36–121 repeats (2). The CAG repeat is inside the *HTT* mRNA-coding region and the expansion lengthens a run of consecutive glutamines within HTT protein. HTT interacts with many proteins and interactions vary depending on whether the repeat expansion is present (6). Numerous functions have been proposed for HTT and it may act as a scaffolding protein (7). The expanded repeat can lead to protein misfolding and aggregation that contributes to disease progression (8).

The link between expression of mutant HTT and HD led to the hypothesis that inhibiting expression of HTT protein might be a productive therapeutic strategy (4). Reducing levels of mutant HTT using duplex RNAs or antisense oligonucleotides leads to reversal of HD symptoms in animal models (9–13). One promising recent result suggests that even a relatively short period of lower mutant HTT levels appears to have a long-term favorable impact on symptoms (13).

Strategies for silencing HTT expression can be either allele selective or non-allele selective. Non-allele-selective approaches reduce levels of both wild-type and mutant HTT expression. One advantage of non-allele-selective approaches is their simplicity—the most efficient silencing agent can be chosen regardless of whether it also reduces expression of the wild-type allele. A disadvantage is that several reports have suggested that HTT plays a role in normal cellular function (14–17). Treating patients with non-allele-selective drugs may decrease the level of wild-type HTT below a threshold necessary for normal function.

Recent reports, however, have demonstrated that sustained repression of wild-type HTT in rhesus striatum (13,18) and mouse brain (13) is well tolerated. While these studies offer hope that relatively simple non-allele-selective approaches have the potential to be useful in patients, concern remains that inhibition of wild-type HTT will have unpredictable and potentially detrimental consequences over long-term treatment. Since mutant HTT is the direct cause of HD, allele-selective inhibition remains an ideal and provides an important alternative for identifying treatments for HD.

One approach towards allele-selective inhibition is to target single-nucleotide polymorphisms (SNPs) associated with expanded repeats (19). It is possible to design duplex RNAs (20) or antisense oligonucleotides (21) that can distinguish SNP differences between the mutant and wild-type HTT alleles. Unfortunately, SNPs vary widely among HD patients and it would be necessary to develop several different nucleic acid drugs to be able to treat a majority of HD patients (22,23). Given the severity of HD and the similarity of each nucleic acid drug (likely to only differ by sequence), developing several drugs and bringing them through multiple similar approval processes may be possible.

Another strategy for achieving allele-selective inhibition is to use compounds that target a variation common to all HD patients—the expanded trinucleotide repeat (24). We hypothesized that selectivity might be achieved because the expanded repeat offers more binding sites for complementary oligonucleotides or possess a hairpin-like structure (25) that is more susceptible to binding. We introduced anti-CAG compounds into cells and discovered that selective inhibition could be achieved by single-stranded antisense oligonucleotides and peptide nucleic acid (PNA) oligomers (26,27).

To identify more potent and selective agents, we attempted to take advantage of efficient gene silencing through RNA interference (RNAi). We tested duplex RNAs that were fully complementary to the expanded trinucleotide repeat and discovered that these compounds were not allele-selective inhibitors (26). We subsequently reasoned that introducing centrally located mismatches might switch the mechanism to that used by miRNAs and present another option for achieving selectivity. This hypothesis proved correct.

Several duplexes with different combinations of mismatched bases exhibited potent inhibition and >30-fold selectivity (28). Krzyzosiak has reported a similar finding (29) and the collective findings have been thoroughly reviewed (30,31). Most recently, we showed that single-stranded RNAs that are chemically modified to function through the RNAi pathway also yield allele-selective inhibition (32).

Robust allele-selective inhibition of HTT expression by duplex RNAs that are partially complementary to CAG repeats is a promising strategy for developing drugs to alleviate HD. The initial reports, however, did little to elucidate the mechanism of inhibition. Here we find that allele-selective inhibition is sensitive to levels of argonaute 2 (AGO2) and GW182/TNRC6 family proteins. Our data are consistent with cooperative inhibition and a mechanism that mimics the action of miRNAs to block translation.

## MATERIALS AND METHODS

### Double-stranded RNAs

Duplex RNAs were purchased from Integrated DNA Technologies (Coralville, IA, USA). Anti-AGO1–4 short interfering RNAs (siRNAs) were used as described (33). Duplex RNAs used for silencing GW182 paralogs were as follows (only guide strand shown): anti-TNRC6A siRNA: 5′-UGAUUGUUAGGCAUCUGGCdTdT-3′; anti-TNRC6B siRNA: 5′-AUUCAUCGCUCGCUUGUCCdTdT-3′; anti-TNRC6C siRNA: 5′-AAGUGGACGUUUGUGGUUCdTdT-3′.

### Cell culture and transfection

Patient-derived fibroblast cell lines GM04281 and GM09197, and mouse striatal precursor cells STHdh Q111/Q7 (CH00096) were obtained from the Coriell Institute (Camden, NJ, USA). The fibroblasts were maintained at 37°C and 5% CO_2_ in Eagle’s Minimal Essential Media (MEM) (Sigma, M4655) supplemented with 10% heat-inactivated fetal bovine serum (Sigma) and 0.5% MEM non-essential amino acids (Sigma). Mouse neuronal cells were cultured at 33°C and 5% CO_2_ in Dulbecco’s Modified Eagle’s Medium (DMEM) (Sigma, D5796) supplemented with 10% heat-inactivated fetal bovine serum (Sigma), 1 mM sodium pyruvate (Sigma) and 0.5% MEM non-essential amino acids (Sigma). Cells were transfected with siRNAs in the presence of lipid RNAiMAX (Invitrogen) according to the previous protocol (26). Cells were typically harvested 3 days after transfection for quantitative PCR (qPCR) or 4 days for protein assay. For double-transfection experiments, the first transfection was performed as described. Media were changed 24 h later, and cells were split into new six-well plate after 72 h of transfection. The second transfection was carried out on the next day. Media were changed again after 24 h, and cells were harvested after 96 h of second transfection for protein analysis.

### Analysis of HTT protein and mRNA

HTT expression was analyzed by western blot analysis and qPCR. HTT protein was separated by SDS–PAGE as described (26–28). Primary antibodies included anti-HTT (MAB2166, Chemicon) and anti-β-actin (Sigma). qPCR was performed on a 7500 real-time PCR system (Applied Biosystems) using iTaq SYBR Green Supermix (Bio-Rad). Data were normalized relative to levels of 18S mRNA. Primer sequences specific for *HTT* are as follows: F 5′-CGACAGCGAGTCAGTGAATG-3′; R 5′-ACCACTCTGGCTTCACAAGG-3′. 18S primers were obtained from Applied Biosystems.

Protein bands were quantified using ImageJ software. The percentage of inhibition was calculated as a relative value to a control sample. Dose fitting curve was generated using GraphPad Prism 4 program by the equation: *y* = 100[1 − *x*^m^/(*n*^m^ + *x*^m^)], where *y* is percentage of inhibition and *x* is the siRNA concentration, *m* and *n* are fitting parameters, where *n* is taken as the IC_50_ value. All the experiments were repeated for at least three times and the error bar is standard deviation.

### *In vitro* cleavage assay

RNA transcript containing *HTT* exon1 with 17 CAG repeats was synthesized by *in vitro* transcription from cloned 5′-end *HTT* fragments and gel purified (27). The RNA transcript was 5′-radiolabeled after dephosphorylation. Two hundred and fifty nanomolars of 5′-phosphorylated siRNA antisense strand and purified recombinant human AGO2 protein (which was generously provided by Dr Qinghua Liu) were pre-incubated at room temperature with 2 µl 10× reaction buffer (0.5 M Tris, pH 7.4, 20 mM MgCl_2_, 5 mM DTT, 2.5 mM ATP, 1 M KCl, 0.5 M NaCl) for 1.5 h. Then the ^32^P-substrate RNA transcript was added and warmed for 1.5 h at 37°C. The RNA was precipitated with 2% LiClO_4_ acetone and separated with 14% acrylamide/7 M urea gel.

### RNA immunoprecipitation

Fibroblasts were seeded in 150 cm^2^ dishes (1400K/dish) and were transfected with duplex RNAs in the next day. Cells (∼90% confluency) were harvested for 72 h. Detached cells were lysed in a buffer [0 mM Tris–HCl pH 7.4, 150 mM NaCl, 2 mM MgCl_2_, 0.5% NP-40, 0.5 mM DTT, protease inhibitor (EDTA-free, Roche) and RNase inhibitor (Promega; 50 U/ml final)] with a volume about three times of the cell pellet size. The mixture was stored in ice for 10 min after thorough mixing. After centrifugation, the supernatant were isolated and stored at −80°C.

Sixty microliters of Protein A/G agarose Plus was incubated with 5 µl of anti-AGO1 (4B8, SAB4200084, Sigma), or anti-AGO2 antibody (015-22031, Wako), anti-GW182 antibody (A302-329A, Bethyl Laboratories) in 1× PBS (pH 7.4) at 4°C with gentle agitation for 2 h. After two washes of 1× PBS, beads were incubated with cell lysate for 2 h at 4°C. The beads were extensively washed with above lysis buffer once, IP wash buffer twice (300 mM NaCl, 3 mM MgCl_2_, 0.5% NP-40 and 20 mM Tris–HCl (pH 7.4) and finally 1× PBS once. The beads were finally eluted with elution buffer (1% SDS, 0.1 M NaHCO_3_ and RNase inhibitor). After proteinase K treatment, RNA extraction and precipitation, samples were treated with recombinant DNase I, followed by reverse transcription. The *HTT* mRNA levels were quantified by qPCR.

STHdhQ111/Q7 murine cells were seeded in 150 cm^2^ dishes (2000K/dish) and transfected at the same time with 50 nM siRNAs in OptiMEM/DMEM with 2% serum (1/1 ratio). Cells were harvested 72 h after transfection. The cytoplasmic fraction was isolated using Cyto lysis buffer [20 mM Tris–HCl (pH 7.4), 150 mM NaCl, 2 mM MgCl_2_ and 0.5% NP-40, 0.5 mM DTT] with proteinase inhibitor and RNase inhibitor. Sixty microliters of Protein A/G agarose Plus was mixed with 5 µg of anti-AGO1 antibody (4B8, SAB4200084, Sigma), or anti-AGO2 antibody (ab57113, Abcam), anti-AGO3 antibody (4B1, gift from Dr Mikiko C. Siomi), anti-AGO4 antibody (5F9.2, 05-967, Millipore) in cyto lysis buffer and the cytoplasmic extracts in 0.5 ml total volume. The mixture was rotated for 3 h at 4°C. After processing, the corresponding cDNA was amplified using allele-specific primers complementary to wild-type or mutant *Htt* mRNA. The primers for wild-type allele: F 5′-CAGGTCCGGCAGAGGAAC-3′ and R 5′-GACTGTGCCACAATGTTTTCA-3′; for mutant allele: F 5′-ACCCGGCCCGGCTGTGGCT-3′ and R 5′-CATTCTGACATCTGACTCCGCATCG-3′. Cyclophilin A is used as internal control, F, 5′-TCGCCGCTTGCTGCA-3′ R, 5′-ATCGGCCGTGATGTCGA-3′. For all the IP data, we first normalized with the IgG, then with a control gene such as GAPDH or cyclophilin A.

## RESULTS

### Experimental design

**Figure 1.**
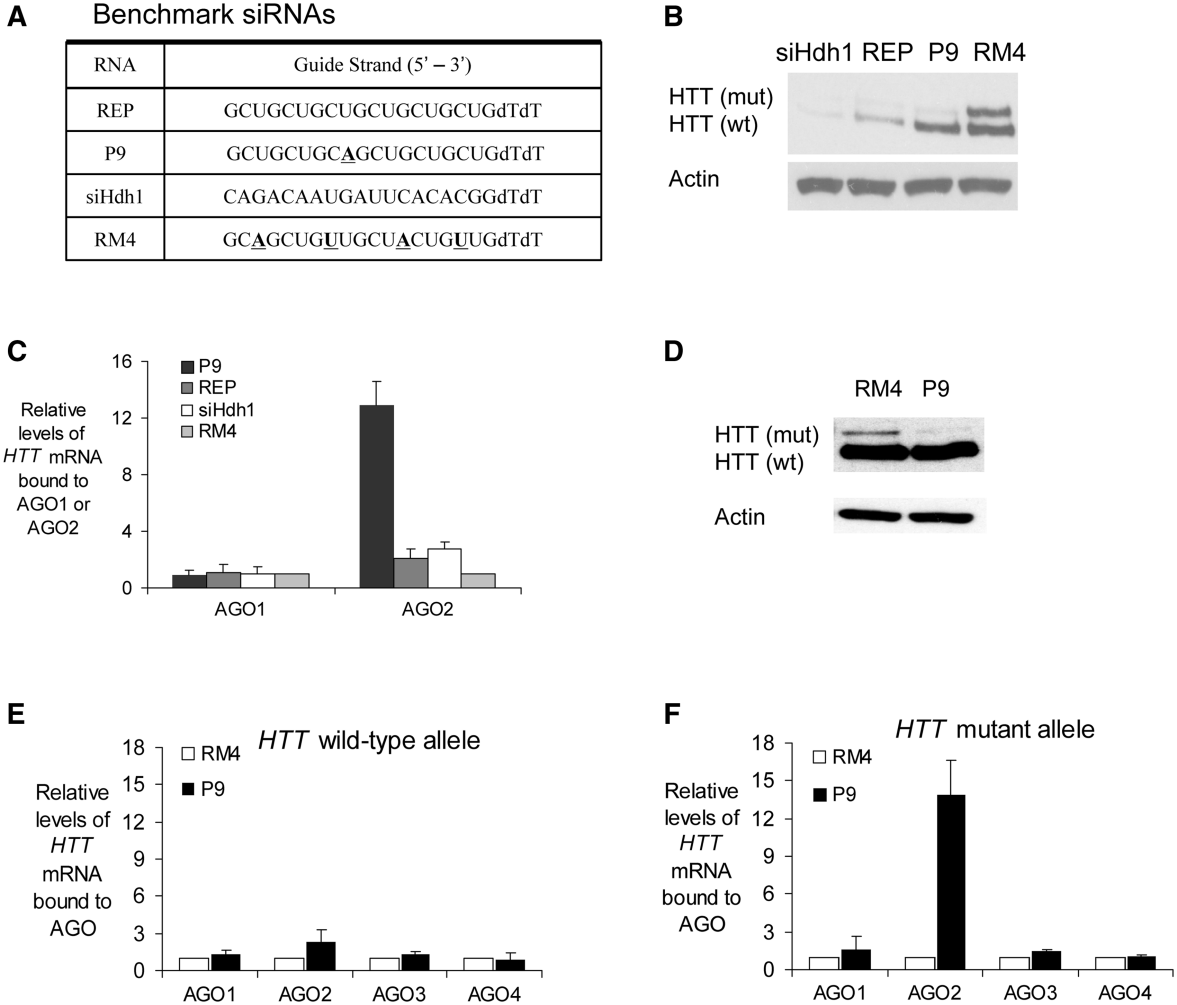
RIP assay examining association of duplex RNAs, AGO proteins and the *HTT* CAG repeat. (**A**) Sequences of benchmark duplex RNAs. (**B**) Allele-selective inhibition of HTT expression by different RNA duplexes (25 nM) in GM04281 patient-derived fibroblast cells. siHdh1 is complementary to a sequence outside of the CAG repeat (34). (**C**) RIP showing association of AGO1 or AGO2 with expanded CAG repeat after transfection of indicated duplex RNAs (25 nM) into GM04281 patient-derived fibroblast cells. RNA levels recovered in the immunoprecipitate were quantified using qPCR. (**D**) Ability of P9 or mismatch containing RM4 duplex RNA (50 nM) to selectively inhibit HTT expression in mouse neuronal cells (STHdhQ111/Q7). RIP showing association AGO1–4 with (**E**) wild-type or (**F**) mutant allele after transfection of P9 or RM4 duplex RNA (50 nM) into mouse neuronal cells. Relative *HTT* mRNA levels were first normalized to the corresponding input samples and then compared to those from samples treated with RM4.

Four duplexes are used throughout this study and were designed as benchmark compounds to explore different approaches towards inhibiting HTT expression (Figure 1A). Duplex siHdh1 (28,34) targets an mRNA sequence outside the CAG repeat that is found in both alleles and inhibits expression with little or no allele selectivity (Figure 1B and Supplementary Figure S1A). Duplex REP is fully complementary to the trinucleotide repeat and also inhibits expression with little or no selectivity (28). Duplex P9 contains a single mismatch relative to the repeat at position 9 and is >30-fold selective for inhibiting mutant HTT expression (28). RM4 contains seed sequence mismatches and is not expected to be active against HTT through the RNAi pathway.

Duplex RNAs were transfected into GM04281 HD patient-derived fibroblasts (17 wild-type/69 mutant repeats, 17Q/69Q) or STHdhQ111/Q7 mouse neuronal precursor cells (7 wild-type/111 mutant repeats) using cationic lipid and samples were harvested for analysis 3 or 4 days after transfection. In some cases, expression of the RNA factors AGO2 or TNRC6A–C was reduced using siRNAs during an initial transfection to test the effects of anti-*HTT* duplexes added in a second transfection.

### Argonaute is selectively recruited to mutant *HTT* mRNA

Because the design of the allele-selective RNA duplexes is identical to the design of duplexes used to silence gene expression through RNAi, we began investigating their mechanism by testing involvement of proteins known to be involved in RNAi. Argonaute (AGO) proteins play a central role in RNAi. There are four AGO proteins in human cells (AGO1–4). AGO2 is the best characterized and is known to promote cleavage of target mRNAs (35,36).

We used RNA immunoprecipitation (RIP) to examine the effect of adding duplex RNAs on recruitment of AGO to the *HTT* trinucleotide repeat. An anti-AGO antibody is use to purify RNAs that associate with AGO. qPCR is then used to detect whether AGO associates with the *HTT* mRNA.

We first used RIP to examine the recruitment of AGO2 to *HTT* mRNA in patient-derived fibroblast cell line GM04281 and observed that mismatch containing RNA P9 recruited AGO2 to *HTT* mRNA (Figure 1C). AGO1 was not recruited. Fully complementary anti-CAG duplex REP did not promote a detectable kinetically stable association between AGO2 and *HTT* mRNA, nor did duplex siHdh1. Both REP and siHdh1 are fully complementary to *HTT* mRNA, and it is likely that they rapidly degrade the RNA target, preventing successful immunoprecipitation by the anti-AGO antibody. In contrast, P9 leaves *HTT* mRNA intact, permitting recovery of *HTT* mRNA by RIP.

For HD fibroblast cells derived from patients, we were unable to use qPCR to distinguish between amplification of the wild-type and mutant alleles. While the two alleles differ in repeat number, distinguishing between was not possible in our hands because of difficulty accurately replicating through the repetitive region. To gain more insight into allele-specific recruitment of AGO, we used STHdhQ7/Q111 mouse neuronal cells. It was possible to independently analyze the amounts of both mutant and wild-type RNA by qPCR because the mutant allele contains human *HTT* exon 1 sequence while the wild-type allele is entirely murine.

Duplex RNA P9 was an allele-selective inhibitor of mutant HTT expression in STHdhQ7/Q111 mouse cells (Figure 1D and Supplementary Figure S1B). RIP showed no evidence that P9 could recruit AGO2 to the wild-type allele (Figure 1E). For the mutant allele, RIP followed by qPCR showed that P9 recruited AGO2 to *Htt* mRNA (Figure 1F). AGO1, AGO3 and AGO4 were not recruited to *Htt* mRNA, consistent with the selectivity observed in Figure 1C. Taken together, these data from human and mouse cells support involvement of AGO2, but not AGO1, AGO3, or AGO4, in the mechanism of allele-selective gene silencing by anti-CAG duplex RNAs.

### Effect of reducing AGO2 expression on allele-selective inhibition

To further probe the role of AGO2 during allele-selective inhibition by anti-CAG duplex RNAs, we used a siRNA pool complementary to *AGO2* mRNA to deplete AGO2 protein from cells (Supplementary Figure S2A). The anti-AGO2 siRNA pool was introduced in an initial transfection followed by anti-*HTT* duplex P9 in a second transfection after 3 days. An important caveat for this experiment is that the silencing of AGO2 is not complete, with ∼20–30% residual expression.

**Figure 2.**
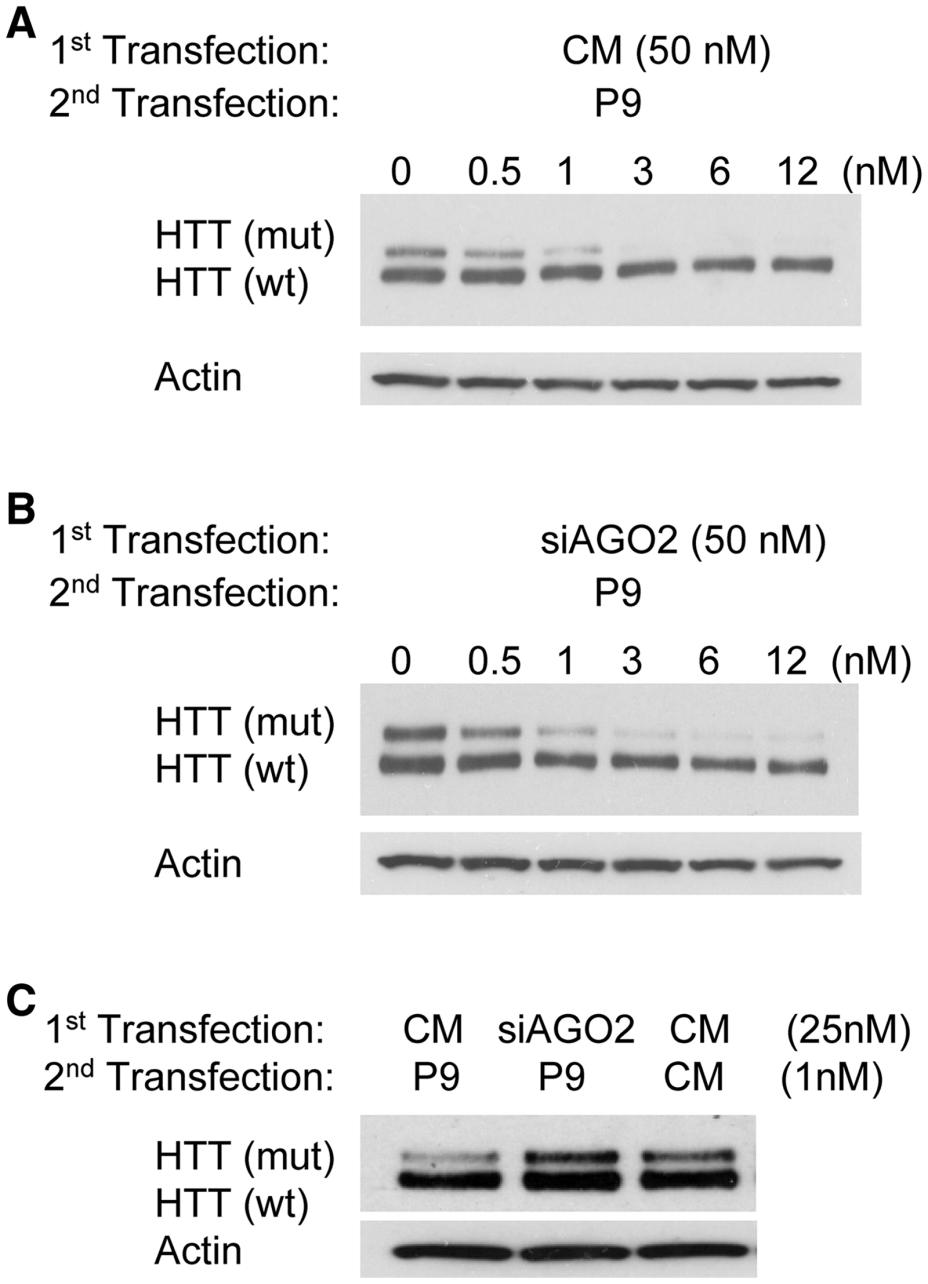
Effect of reducing AGO2 expression on allele-selective inhibition of mutant HTT in GM04281 fibroblast cells. In an initial transfection levels of cellular AGO2 were reduced by transfection with 50 nM siAGO2 pool designed to target AGO2 mRNA. For comparison transfection was also done with 50 nM non-complementary duplex CM. (**A**) Effect of adding a non-complementary RNA duplex CM (50 nM) during the initial transfection followed by RNA P9 in a second transfection. (**B**) Effect of adding anti-AGO2 duplex RNA pool on inhibition of HTT over a broad range of concentrations of duplex RNA P9. (**C**) Reversal of P9-mediated allele-selective inhibition under a narrow range of concentrations after AGO2 knockdown using 25 nM anti-AGO2 duplex RNA pool and 1 nM RNA P9.

When non-complementary control duplex CM was added during the first transfection, duplex P9 remained a potent and highly allele-selective inhibitor of mutant HTT expression (Figure 2A). We examined reduction of AGO2 levels. In view of the observed recruitment of AGO2 to *HTT* mRNA, we were surprised to observe that reduction of AGO2 expression by addition of the anti-AGO2 siRNA pool during the first transfection also had little effect on allele-selective inhibition (Figure 2B and Supplementary Figure S2B). Reduction of AGO1, AGO3 and AGO4 had a similar lack of impact (Supplementary Figure S2C and D). Careful titration of experimental conditions eventually identified concentrations of anti-AGO2 siRNA and duplex P9 that, when used together, could reverse allele-selective silencing (Figure 2C and Supplementary Figure S2E). The narrow window where reducing AGO2 has an effect emphasizes that relatively small amounts of residual AGO2 are sufficient to promote allele-selective inhibition of mutant HTT of duplex P9.

### Reducing AGO2 levels transforms non-allele-selective inhibition

**Figure 3.**
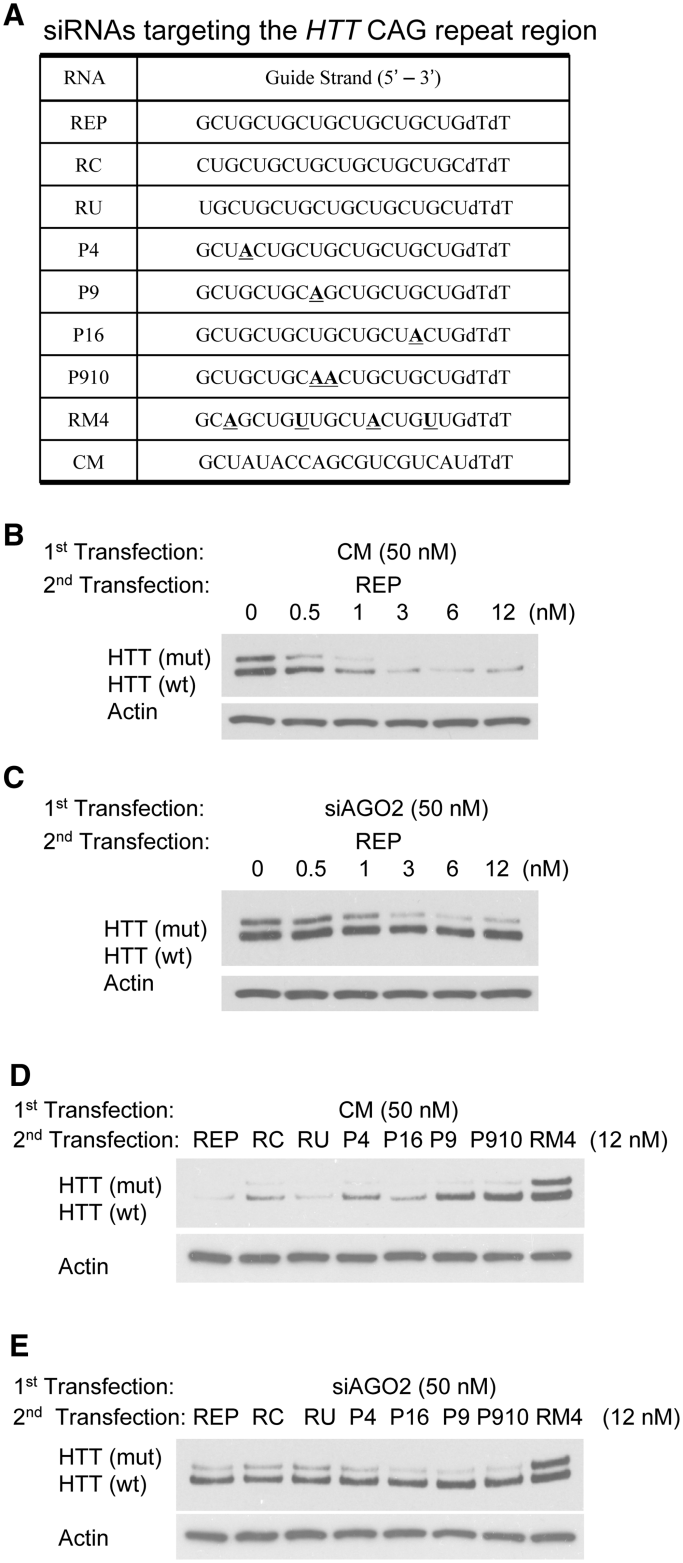
Reducing AGO2 expression converts duplex RNA REP and other previously non-allele-selective duplexes into allele-selective silencing agents. Experiments were performed in GM04281 fibroblasts. In an initial transfection levels of cellular AGO2 were reduced by transfection with 50 nM siAGO2 designed to target AGO2 mRNA. For comparison transfection was also done with 50 nM non-complementary duplex CM. (**A**) Sequences of duplex RNAs. (**B**) Effect of adding a non-complementary RNA duplex (CM, 50 nM) during the initial transfection on silencing by duplex RNA REP. (**C**) Effect of reducing AGO2 using an anti-AGO2 duplex RNA (50 nM) of duplex RNA REP. (**D**) Effect of adding a non-complementary RNA duplex (CM) during the initial transfection on silencing by several different non-allele-selective RNAs (12 nM). (**E**) Effect of reducing AGO2 expression, using anti-siAGO2 duplex RNA (50 nM), on the action of previously non-allele-selective RNAs.

We next examined the impact of reducing levels of AGO2 on inhibition of HTT expression by duplex RNA REP and other non-allele-selective duplex RNAs (Figure 3A). In contrast to the narrow window for achieving detectable effects of AGO2 depletion on allele-selective inhibition of mutant HTT, reduced expression of AGO2 over a wide range of concentrations caused REP to change from a non-allele-selective inhibitor (Figure 3B and Supplementary Figure S3A) to an efficient allele-selective agent (Figure 3C and Supplementary Figure S3A). We tested several other non-allele-selective inhibitors (Figure 3D and Supplementary Figure S3B) with complementarity to the CAG repeat and found that they all became selective when levels of AGO2 were reduced (Figure 3E and Supplementary Figure S3C).

These data are consistent with the conclusion that reduced levels of AGO2 remain sufficient to support inhibition of mutant HTT expression. Reduced levels of cellular AGO2 are not, however, adequate to inhibit expression of wild-type HTT. Under conditions of limiting AGO2, previously non-allele-selective duplex RNAs become allele selective. The varying dependence of allele selectivity on AGO2 concentration suggests that the mechanisms of recognition of the mutant and wild-type alleles differ significantly.

### Allele-selective inhibition requires GW182 paralogs

GW182 (TNRC6A) is a critical factor in translational silencing by miRNAs (37–42). GW182 has three paralogs in human cells, TNRC6A, TNRC6B and TNRC6C (38). GW182/TNRC6A–C can bind multiple AGO proteins (40), providing the potential for synergistic or cooperative binding of multiple species.

**Figure 4.**
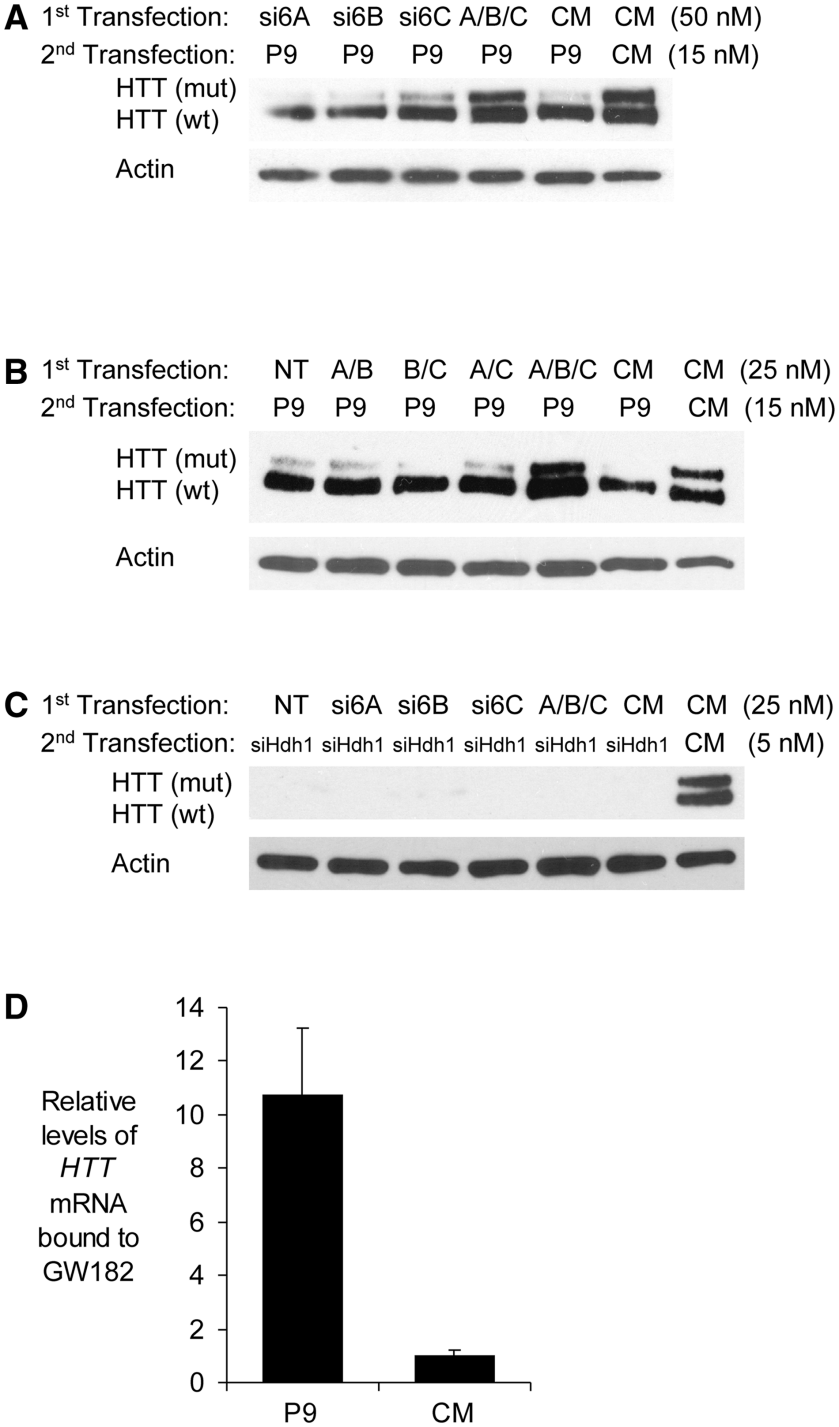
Effect of reducing GW182 paralogs alone or in combination on allele-selective inhibition of HTT expression by duplex RNA P9 in GM04281 fibroblast cells. (**A**) Inhibiting expression of all three GW182/TNRC6A paralogs reverses allele-selective inhibition. When si6A, si6B and si6C were added together, each was present at 16.6 nM. (**B**) Inhibiting combinations of any two GW182/TNRC6A paralogs does not reverse allele-selective inhibition. When two siRNAs were used together, their total concentration was 25 nM. (**C**) Inhibiting expression of all three GW182/TNRC6A paralogs alone or in combination does not block silencing by siRNA siHdh1. When three siRNAs were used together, their total concentration was 25 nM. (**D**) RIP showing recruitment of GW182 to *HTT* mRNA after addition of duplex RNA P9 or non-complementary control CM (50 nM). A or si6A: siRNA targeted TNRC6A. B or si6B: siRNA targeted TNRC6B. C or si6C: siRNA targeted TNRC6C.

To determine the role of GW182 family proteins in allele-selective inhibition of mutant human HTT, we inhibited expression of TNRC6A, TNRC6B or TNRC6C individually using siRNAs that target their mRNAs (Supplementary Figure S4A and B). Individual reduction of the GW182 paralogs had no effect on inhibition of HTT (Figure 4A). When we simultaneously inhibited all three paralogs, allele-selective inhibition of mutant HTT was reversed, suggesting that GW182 expression was important but that the paralogs had overlapping functions. Subsequent attempts to inhibit any two paralogs in combination also failed to reverse allele-selective inhibition (Figure 4B and Supplementary Figure S4C). While inhibition of all three TNRC6 paralogs blocked allele-selective inhibition of HTT expression, blocking expression of all three paralogs had no effect on non-allele-selective inhibition by a duplex RNA siHdh1 complementary to an mRNA sequence outside of the CAG repeat (Figure 4C and Supplementary Figure S4D). RIP using an anti-TNRC6A antibody demonstrated that addition of anti-CAG duplex RNA caused recruitment of TNRC6A to the *HTT* mRNA (Figure 4D).

These experiments indicate that the GW182 family proteins are necessary for allele-selective silencing and can substitute for one another. Full expression of just one protein is sufficient to support selective repression of mutant HTT. Silencing by an RNA targeting the trinucleotide repeat is much more sensitive to TNRC6 expression than is silencing by an RNA targeting outside the CAG repeat, consistent with differing mechanisms of action.

### Allele-selective inhibition may involve cooperative binding

The repetitive region within a mutant *HTT* mRNA with 69 repeats has the potential to bind up to 9–10 twenty-base-long oligomers. A wild-type mRNA with 17 repeats, in contrast, is predicted to bind no more than two. It is possible that binding of multiple oligomers at adjacent sites might lead to cooperative inhibition and contribute to allele-selective recognition of expanded mutant repeat regions. Cooperative binding would lower the concentration of AGO needed to produce an effect. This outcome would be consistent with our experimental observation that reduced AGO2 levels maintain inhibition of mutant HTT expression while reducing inhibition of wild-type HTT.

**Figure 5.**
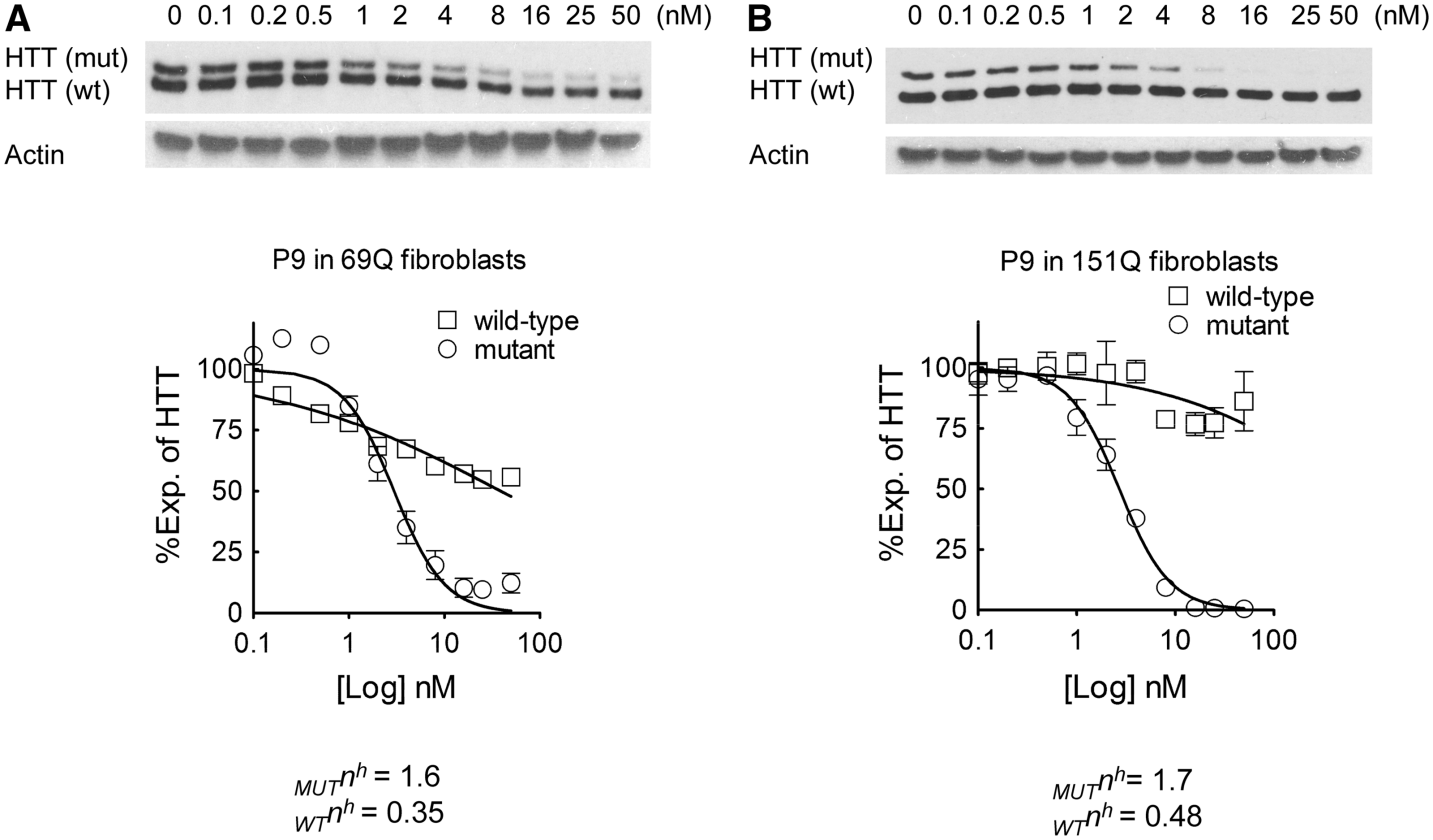
The dependence of silencing mutant HTT expression by siRNA P9 suggests cooperative binding. siRNA P9 was transfected at increasing concentrations in HD patient fibroblasts with mutant alleles containing (**A**) 69 CAG repeats in GM04281 cells, or (**B**) 151 CAG repeats in GM09197 cells.

To determine whether allele-selective inhibition is due to cooperative binding of expanded *HTT* mRNA, we examined inhibition of mutant HTT by duplex P9 in GM04281 fibroblasts (69Q/17Q) over a wide range of concentrations. After fitting the data to the Hill equation (43), we obtained Hill coefficients, *n*^h^, of 1.6 and 0.35 for inhibition of mutant and wild-type HTT expression, respectively (Figure 5A). We also examined inhibition in fibroblast cell line GM09197 containing 151 mutant repeats and 21 wild-type repeats (151Q/21Q) and found Hill coefficients, *n*^h^, of 1.7 and 0.48 for inhibition of mutant and wild-type HTT expression, respectively (Figure 5B). These data are consistent with cooperative inhibition and suggest that association of duplex RNA with the expanded mutant repeat is likely to involve multiple binding events. Recently, we have also reported observing cooperative binding by single-stranded siRNAs that function through the RNAi pathway (32).

### Mismatches are not tolerated adjacent to trinucleotide repeat

Duplexes complementary to the expanded mutant CAG repeat can recognize more than one site within the repeat. In contrast to the potential for binding multiple RNAs within the expanded repeat, target sequences outside the repeat are unique and can bind only one silencing RNA. If cooperativity were contributing to allele-selective inhibition of HTT, one prediction would be that duplexes complementary to the CAG repeat would tolerate mismatched bases more than duplexes that are complementarity to non-repeat sequences. The potential for cooperative interactions might overcome the reduced binding affinity of the mismatched bases.

**Figure 6.**
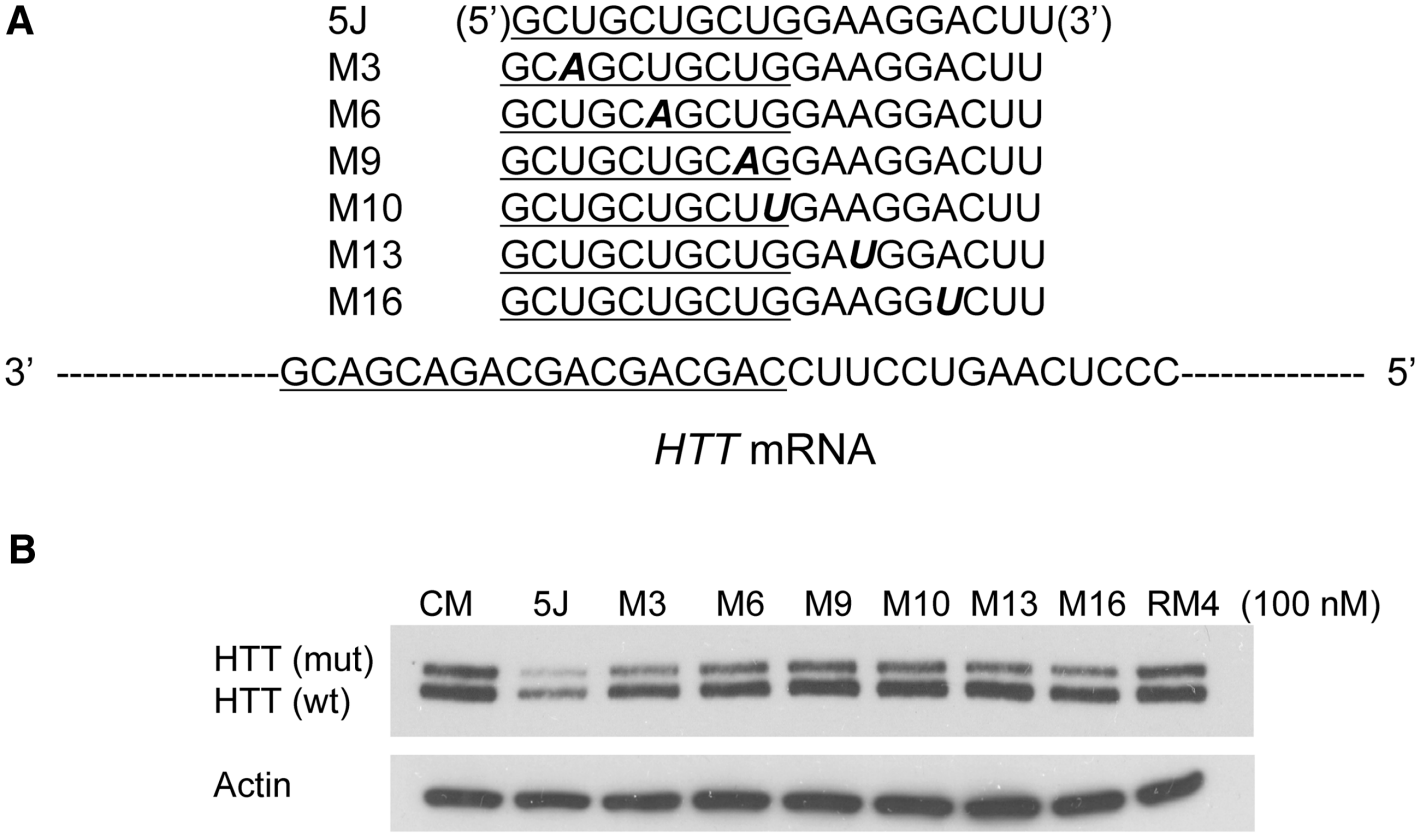
Effect of introducing mismatches into a duplex RNA that targets the 5′-junction between the CAG repeat and the remainder of *HTT* mRNA in GM04281 fibroblasts. (**A**) Sequences of RNAs targeting the *HTT* 5′ CAG junction relative to the complementary region of *HTT* mRNA. (**B**) Inhibition of HTT by RNAs (100 nM) targeting the 5′-junction in GM04281 cell line.

We tested this prediction by introducing mismatched bases into duplex RNA 5 J targeting the junction between the CAG repeat and the 5′-portion of *HTT* mRNA (Figure 6A). The target sequence for RNA 5J is unique, allowing just one RNA to bind, while remaining close to the CAG repeat. While duplexes complementary to the CAG repeat tolerated as many as four mutations outside the seed sequence region (28), inhibition was abolished when just one mismatched base was present in duplexes targeting the 5′-junction sequence (Figure 6B and Supplementary Figure S5). This result is consistent with the possibility that cooperative binding can compensate for weaker recognition by mismatch-containing duplexes.

### Mismatch-containing anti-CAG RNAs do not cause transcript cleavage

AGO2 is the only AGO variant in human cells capable of efficiently cleaving mRNA. AGO2-mediated cleavage is disrupted by the presence of mismatched bases between a small RNA guide strand and its mRNA target at positions 9 and 10 (44). We hypothesized, therefore, that mismatch-containing duplexes would not cause cleavage of their targets within the *HTT*-expanded repeat.

**Figure 7.**
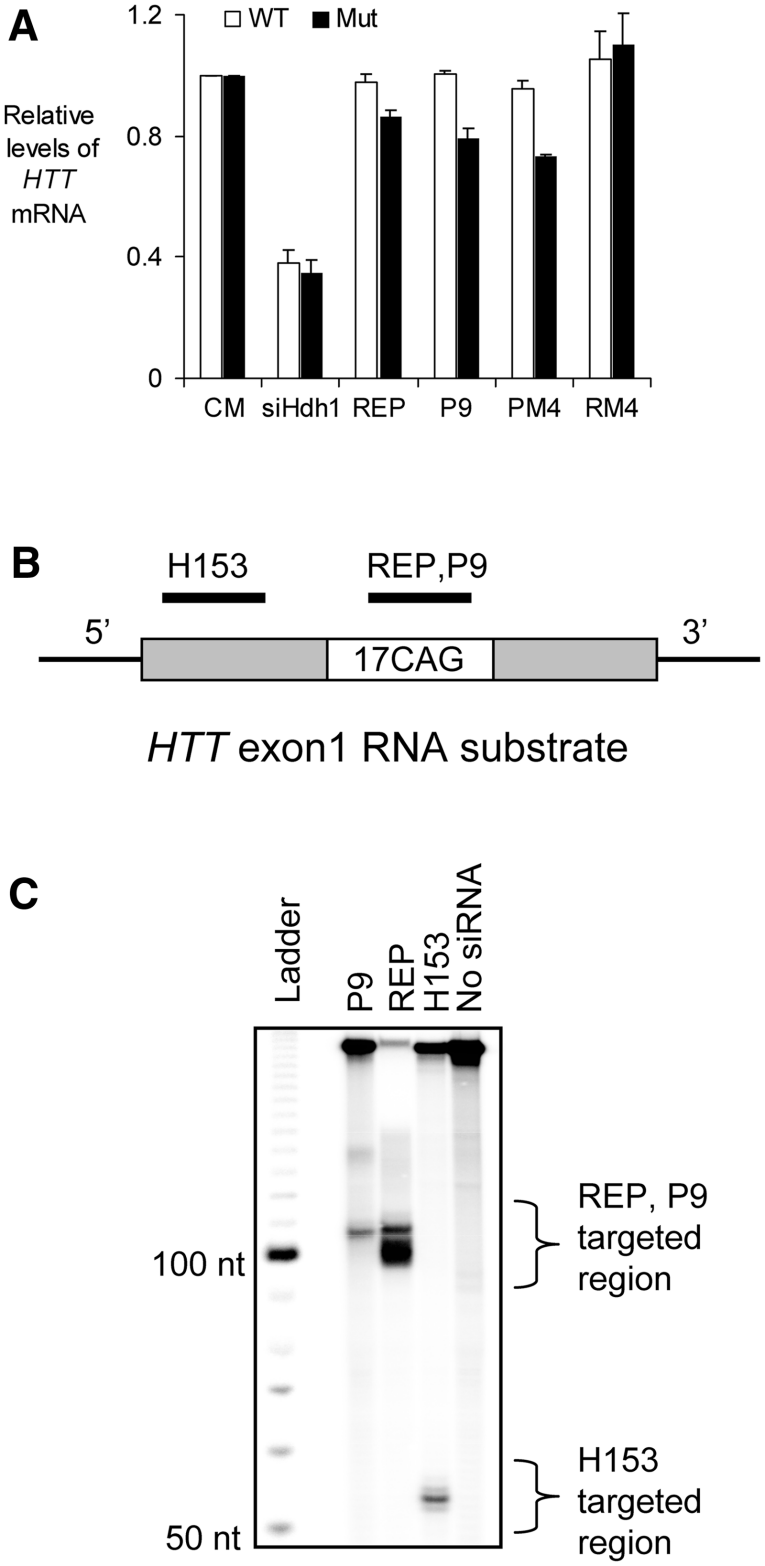
Measurement of RNA levels and *In vitro* RNA cleavage assay using recombinant hAGO2 protein. (**A**) Effect of duplex RNAs on *HTT* mRNA levels in STHdhQ111/Q7 murine neuronal cells. (**B**) Schematic drawings of the 5′-end radiolabeled *HTT* exon 1 substrate RNA containing 17 CAG repeat and the siRNA targeted regions. (**C**) siRNA-directed target RNA cleavage using recombinant hAGO2. siRNA H153 is a positive control. The cleaved product is indicated on the right.

To test this hypothesis, we examined the effect of transfecting duplex P9 into murine striatal precursor cell line STHdhQ111/7. We used STHdhQ111/7 cells because they allow detection and measurement of both the mutant and wild-type *HTT* mRNA. Duplex P9 did not significantly reduce levels of either allele, not did duplex PM4 (28) that contains four mismatches and is a potent and allele-selective inhibitor of mutant HTT expression (Figure 7A). In contrast, duplex siHdh1 that was complementary to a sequence outside the CAG repeat reduced both alleles.

To further test whether these mutations also disrupt cleavage of mRNA upon AGO2-mediated recognition of *HTT* mRNA, we performed an *in vitro* cleavage assay (Figure 7B and C). In this assay, purified recombinant human AGO2 was pre-incubated with siRNA guide strand and then mixed with a radiolabeled exon 1 *HTT* mRNA transcript that had been transcribed *in vitro* and contained 17 CAG repeats. A 17-repeat substrate was used because longer repeat constructs were difficult to synthesize. We observed that fully complementary REP caused cleavage of the radiolabeled substrate, while RNA P9 yielded much less cleavage of repeat RNA. Together with our observation that mismatch-containing anti-CAG RNAs do not reduce *HTT* mRNA levels, these data suggest that resistance to cleavage is an important factor contributing to allele-selective inhibition.

## DISCUSSION

Antisense oligonucleotides and duplex RNAs are a realistic approach for drug therapy (45). One antisense oligonucleotide, fomivirsen, has been approved as a drug. Another, mipomersen, has been submitted for FDA review after several favorable Phase III trials for reducing cholesterol levels after systemic administration (46). Because nucleic acids can control specific disease genes, they offer a promising approach to treating neurological disease. One oligonucleotide is in Phase I trials for amyotrophic lateral sclerosis (47) and several pre-clinical studies have shown gene silencing in primate brains (48).

HD is caused by a dominant mutation in a specific gene, *HTT*, making it an ideal target for drug development strategies using siRNAs or antisense oligonucleotides (4). Our laboratory has investigated inhibition of HTT by duplex RNAs complementary to the expanded CAG repeat within *HTT* mRNA (28). These compounds are both potent and allele selective, making them good starting points for drug development. The duplex RNAs also inhibit expression of the mutant-expanded CAG repeat allele of *Ataxin-3* (49), the cause of Machado Joseph Disease, suggesting that one compound might be able to treat multiple hereditary disorders.

**Figure 8.**
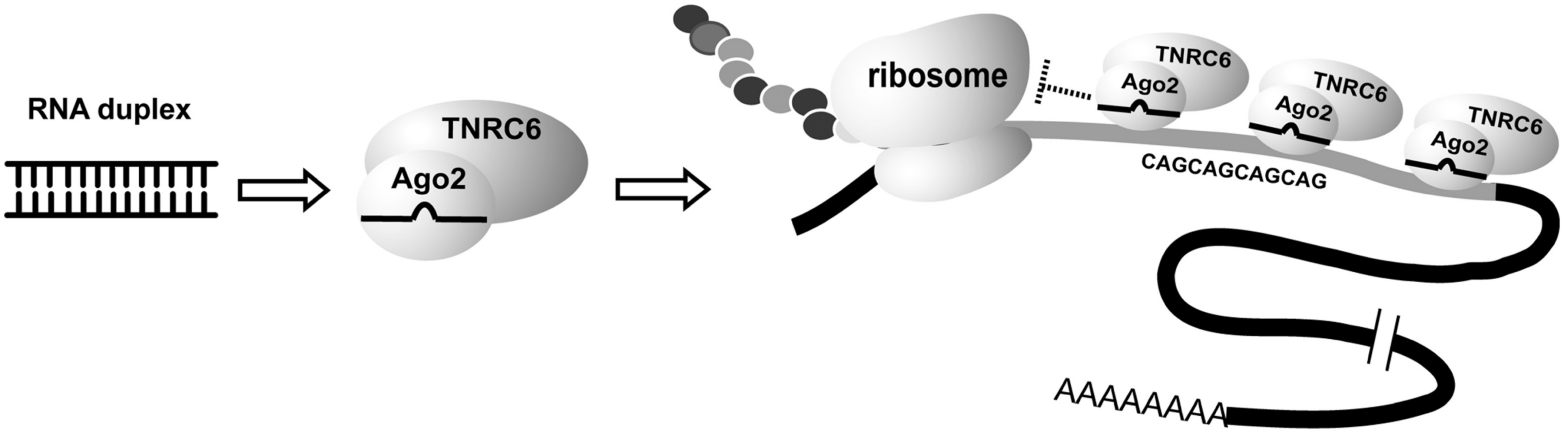
Scheme for translational repression of mutant *HTT* mRNA by mismatch-containing duplex RNAs that target CAG repeats. An RNA duplex enters cells and associates with AGO2 and TNRC6 family proteins. The AGO2–TNRC6–small RNA complex recognizes the expanded repeat at more than one site, forming a block that is strong and stable enough to prevent translation.

Our data suggest that the first step in the mechanism of allele-selective silencing is AGO2-mediated recognition of *HTT* mRNA (Figure 8). Because the expanded repeat offers multiple binding sites, more than one anti-CAG RNA complex can bind to each mRNA repeat simultaneously. GW182/TNRC6, a protein family that interacts with AGO2, is also recruited to the expanded CAG repeat. GW182 and its paralogs are known to have the capacity to bind multiple AGO proteins (40) and we speculate that the sharp dependence on GW182 expression (Figure 4) may reflect bridging of AGO complexes and promoting cooperative interactions. Because mismatches are introduced into the anti-CAG duplex RNA, the ability to induce cleavage of mRNA is greatly reduced and *HTT* mRNA remains intact. Cooperative binding of multiple mismatched RNAs may be necessary to create a complex that binds strongly enough to disrupt translation of the mutant allele.

AGO2-assisted recognition at the CAG repeat is efficient and gene silencing continues to occur even when AGO2 protein levels are reduced. In contrast, silencing of the wild-type allele is much more sensitive to AGO2 levels. These data support the hypothesis that the RNA–AGO2 complex is involved in cooperative interactions within the expanded trinucleotide repeat. It is possible that GW182 may be involved as well. Because interactions are cooperative, binding to adjacent sites is more robust and becomes less reliant on the cellular pool of AGO2 and less responsive to decreased AGO2 levels.

The mechanism of allele-selective inhibition by anti-CAG RNAs is reminiscent of the action of miRNAs. Unlike anti-CAG duplexes, most known target sites for miRNAs are within 3′-untranslated regions. However, like anti-CAG duplexes, there are often multiple adjacent binding sites (50). When inhibition by miRNAs targeting within coding regions is observed, the targets are often rich in repeats with the potential for binding more than one miRNA (51).

Cell-based studies by using plasmids with potential miRNA-binding sites varying in number and spacing suggested cooperative action (52,53) by AGO 1, AGO3 or AGO4 to achieve gene silencing (53). Interestingly, AGO2 and catalytically inactive AGO2 did not yield cooperative silencing when fully complementary duplexes were used (53), but did show cooperative silencing when duplexes contained central mismatches. The authors suggest that AGO2 may form different associations depending on whether an exact match or bulged duplex is formed, and it is possible that similar considerations contribute to the allele selectivity of mismatch-containing duplexes that we observed.

Our data show that allele-selective duplex RNAs function through the RNAi pathway. Development of duplex RNAs as candidates for drug development will require testing the impact of the chemical modifications needed to optimize biodistribution and stability *in vivo*. Cooperativity may explain the potency of our compounds and chemical modifications should be chosen to retain or enhance the potential for cooperative interactions. This improved understanding of mechanism should facilitate prioritizing the selection of duplex RNAs and expedite drug design and development.

## SUPPLEMENTARY DATA

Supplementary Data are available at NAR Online: Supplementary Figures 1–5.

## FUNDING

Work in the Corey Laboratory was supported by the National Institutes of Health (NIH) [NIGMS 73042]; an award from the McKnight Foundation for Neuroscience; Cure Huntington's Disease Initiative (CHDI) Inc.; Robert A. Welch Foundation [I-1244]. Funding for open access charge: NIH.

*Conflict of interest statement*. We have filed a patent application related to the discovery that mismatch-containing RNAs inhibit HTT expression.

## Supplementary Material

Supplementary Data

Supplementary Data
