# Supplementary material for: Mechanism of allele-selective inhibition of huntingtin expression by duplex RNAs that target CAG repeats: function through the RNAi pathway
Source: Nucleic Acids Res. 2012 Oct 5;40(22):11270–80. doi: 10.1093/nar/gks907 (PMC3526262; doi:10.1093/nar/gks907)
Supplement: Supplementary Data [file supp_40_22_11270__index.html]

Mechanism of allele-selective inhibition of huntingtin expression by duplex RNAs that target CAG repeats: function through the RNAi pathway — Mechanism of allele-selective inhibition of huntingtin expression by duplex RNAs that target CAG repeats: function through the RNAi pathway — Supplementary Data 

# Mechanism of allele-selective inhibition of huntingtin expression by duplex RNAs that target CAG repeats: function through the RNAi pathway

## Supplementary Data

files

**Files in this Data Supplement:**

- Supplementary Data - pdf file
