# Supplementary material for: Mechanism of allele-selective inhibition of huntingtin expression by duplex RNAs that target CAG repeats: function through the RNAi pathway
Source: Nucleic Acids Res. 2012 Oct 5;40(22):11270–80. doi: 10.1093/nar/gks907 (PMC3526262; doi:10.1093/nar/gks907)

Figure S1. Averaged HTT protein levels after treated with siRNAs. A). Quantitation of gel images in figure **1b**. Effects of HTT expression after treated with 25 nM of siRNAs in HD fibroblasts (GM04281, CAG 69/17). **B**) Quantitation of gel images in figure **1d**. Effects of HTT expression after treated with 50 nM of siRNAs in mouse neuronal progenitors cells STHdhQ111/Q7.

A

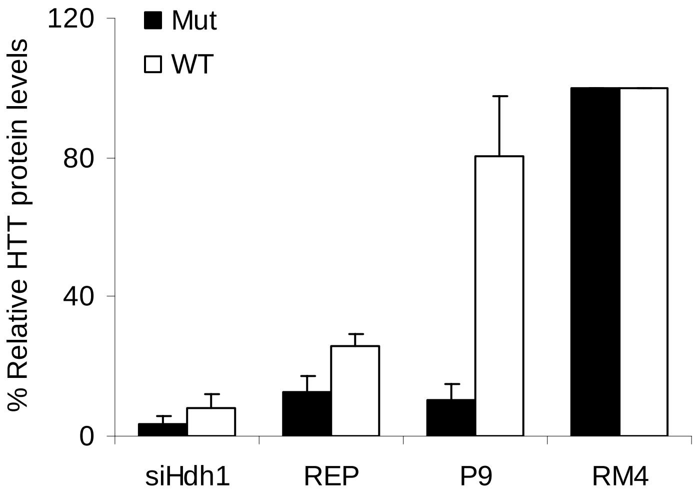

B

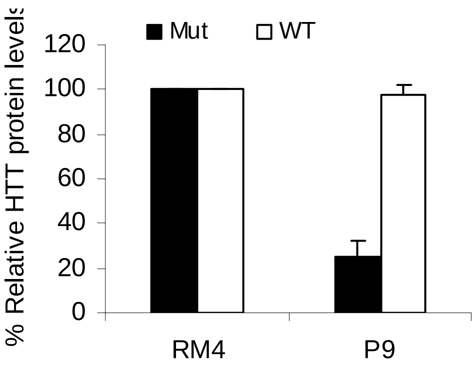

Figure S2. Effects of reducing AGO2 expression to allele-selective inhibition of mutant HTT by duplex RNA P9 in in HD patient fibroblasts (GM04281, CAG 69/17).
A) AGO2 protein levels were significantly reduced by anti-AGO2 siRNAs. **B**) Quantitation of gel images in figure **2a,b**. Dose curves of CM/P9 treated are in black and dotted lines. Dose curves of siAgo2/P9 treated are in red lines. **C**) AGO1,3,4 expressions were effectively reduced by siRNAs.

A

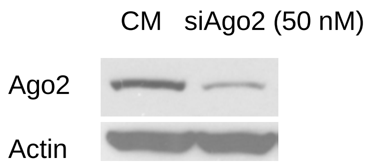

B

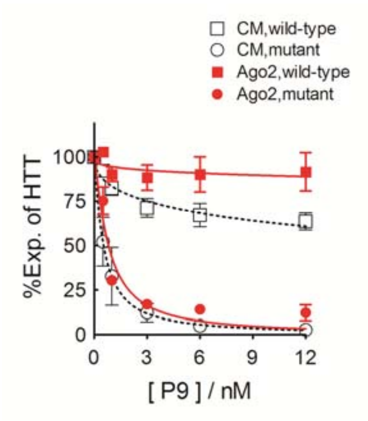

C

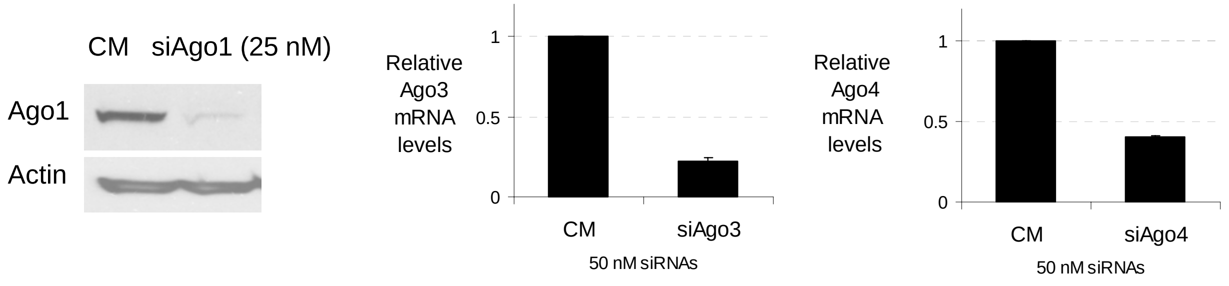

Figure S2 (continued). Effects of reducing AGO1,3,4 expressions to allele-selective inhibition of mutant HTT by duplex RNA P9 in HD patient fibroblasts (GM04281, CAG 69/17). D). Effects of reducing AGO1,3,4 expressions over increased concentrations of RNA P9. Representative western blot images were showed. Figures below are averaged dose curves. Curves of CM/P9 treated are in black and dotted lines. Dose curves of siAgo/P9 treated are in red lines. **E**). Quantitation of gel images in figure **2c**.

D

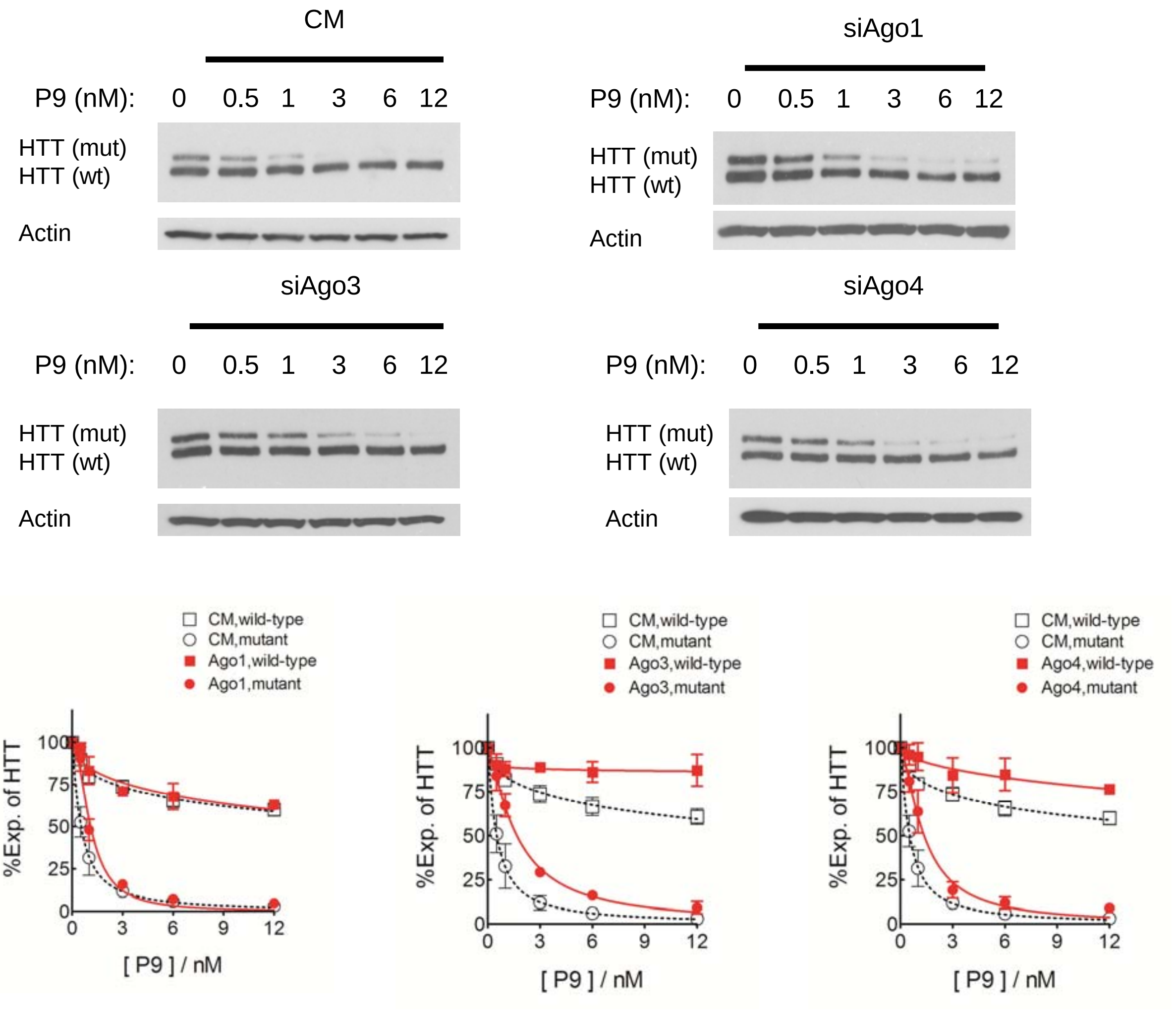

E

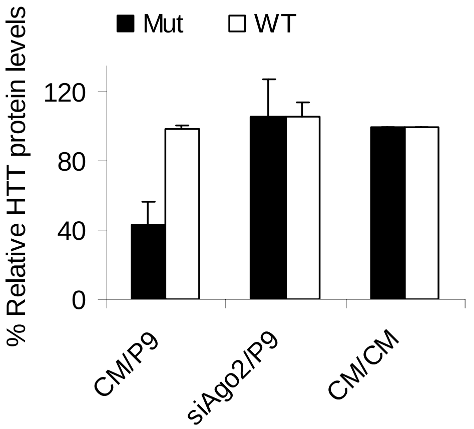

Figure S3. Quantitation of HTT expressions in Fig.3. Effect of reducing Ago2 levels to the function of non-selective siRNAs. A) Averaged dose curves in figure **3b,c**. Curves of CM/REP treated are in black and dotted lines. Dose curves of siAgo2/REP treated are in red lines. **B**) Quantitation of gel images in figure **3d**. **C**) Quantitation of gel images in figure **3e**. All experiments were done in HD patient fibroblasts (GM04281, CAG 69/17).

A

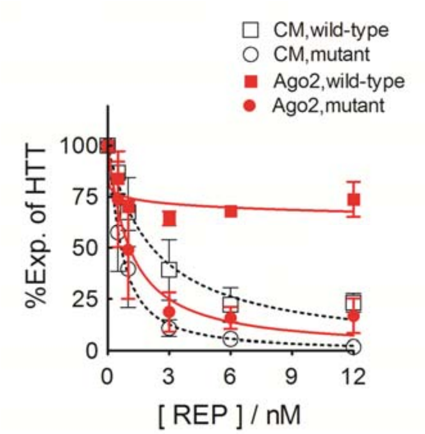

B

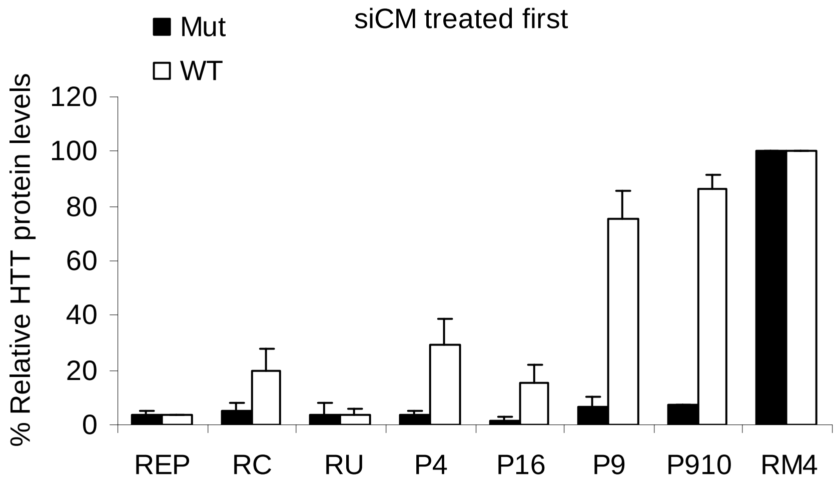

C

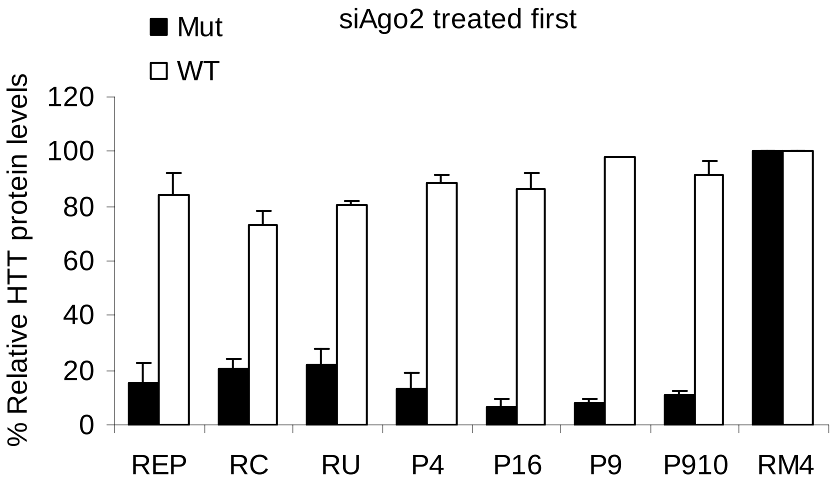

Figure S4. siRNAs targeting TNRC6A, 6B, or 6C efficiently and specifically knock down the expression of only the targeted isoform. si6A: siRNA targeting TNRC6A (GW182); si6B: siRNA targeting TNRC6B; si6C: siRNA targeting TNRC6C; NT: no treatment. CM: noncomplementary RNA duplex. **A).** QPCR analysis of TNRC6A/B/C mRNA levels. **B).** Representative western blot images.

A

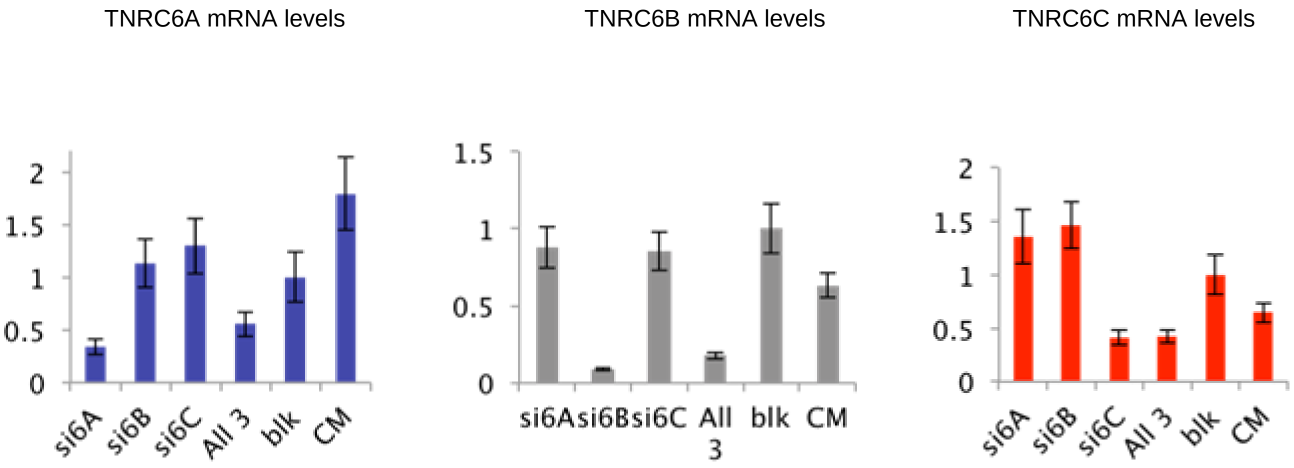

B

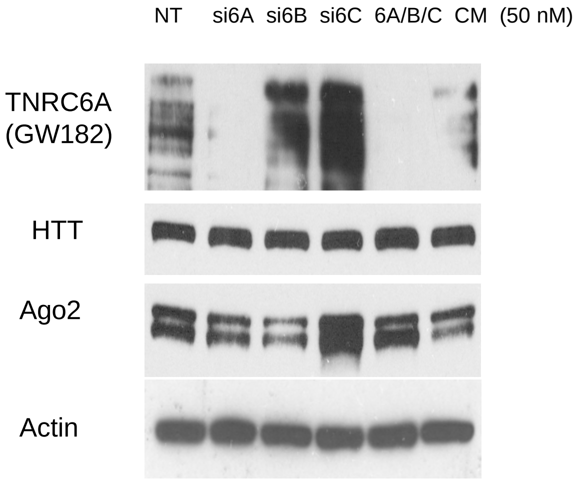

Figure S4 (continued). Quantitation of HTT expressions in Fig.4. C) Effect of reducing TNRC6A,B,C levels to the function of siRNA/P9. Quantitation of gel images in figure **4a**,**b**. **D**) Effect of reducing TNRC6A,B,C levels to the function of siHdh1. Quantitation of gel images in figure **4c**. All experiments were done in HD patient fibroblasts (GM04281, CAG 69/17).

C

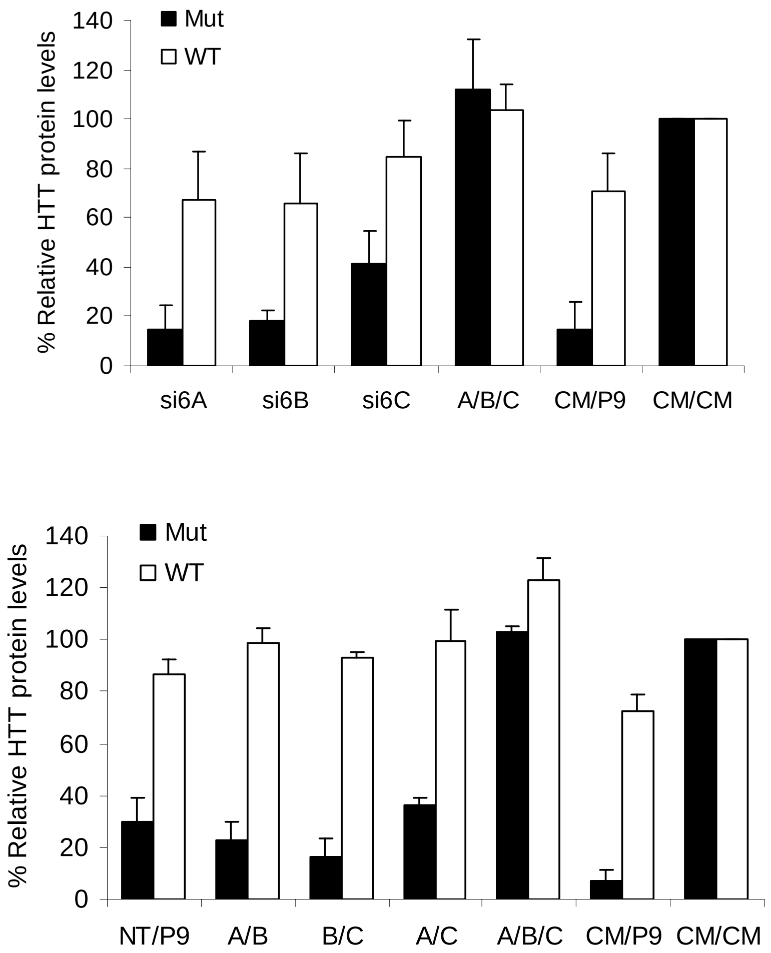

D

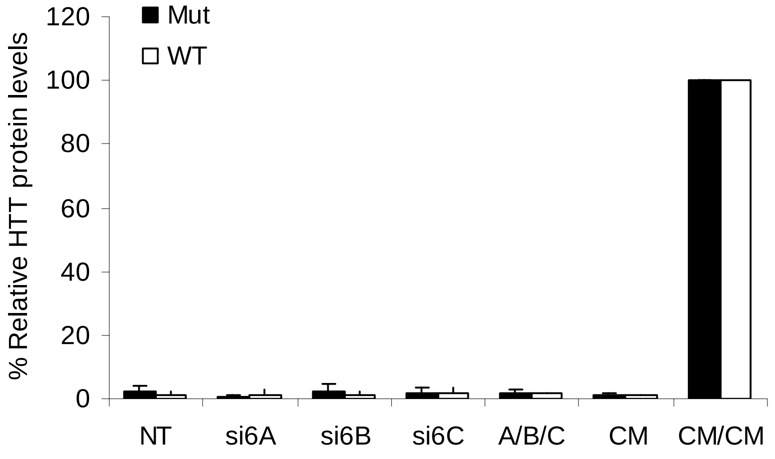

Figure S5. Quantitation of HTT expressions in Fig.6. Effects of treating siRNAs targeting the HTT 5'-CAG junction to HTT expression. All experiments were done in HD patient fibroblasts (GM04281, CAG 69/17).

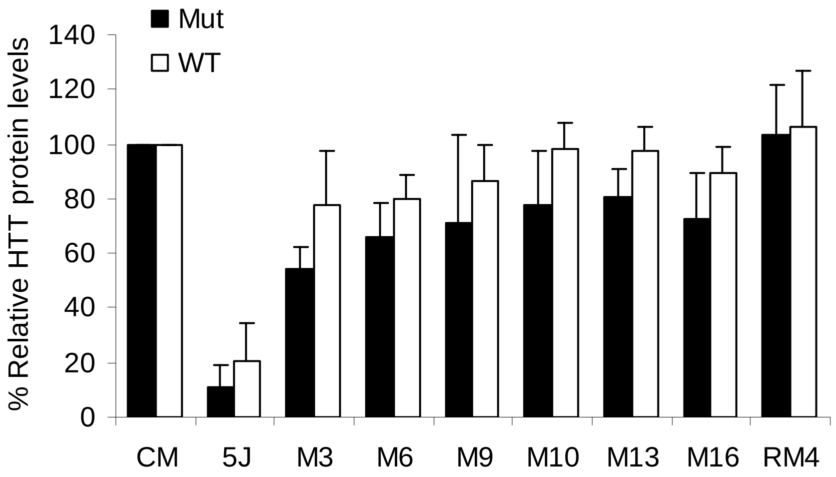

Supplement: Supplementary Data [file supp_gks907_nar-01810-h-2012-File010.pdf]
